# Comment on: Machine Learning for Understanding and Predicting Injuries in Football

**DOI:** 10.1186/s40798-024-00745-1

**Published:** 2024-07-28

**Authors:** Garrett S. Bullock, Patrick Ward, Gary S. Collins, Tom Hughes, Franco Impellizzeri

**Affiliations:** 1 Department of Orthopaedic Surgery and Rehabilitation, Wake Forest University School of Medicine, Winston‑Salem, NC USA; 2 Department of Biostatistics and Data Science, Wake Forest School of Medicine, Winston-Salem, NC USA; 3 Centre for Sport, Exercise and Osteoarthritis Research Versus Arthritis, University of Oxford, Oxford, UK; 4 Seattle Seahawks, Seattle, WA USA; 5 Centre for Statistics in Medicine, University of Oxford, Oxford, UK; 6 Department of Health Professions Institute of Sport, Manchester Metropolitan University, Manchester, UK; 7 Institute of Sport, Manchester Metropolitan University, Manchester, UK; 8 School of Sport, Exercise, and Rehabilitation, University of Technology Sydney, Sydney, Australia

Dear Editor,

We recently read the article titled “Machine Learning for Understanding and Predicting Injuries in Football” in Sports Medicine – Open [1]. Given that injury prediction is an emerging topic within sport, the increasing interest and excitement towards complex machine learning algorithms within this space is a cause for concern when fundamental principles of prediction model development are not followed. As such, we feel the need to intervene and highlight methodological and conceptual inaccuracies.

The models presented in this paper were deemed by the authors to be “quite sound” [1]. However, this is not true, as recently highlighted in the systematic review in Sports Medicine [2]. All of these models were included in this systematic review, and after evaluation with the established Prediction Model Risk of Bias Assessment Tool (PROBAST) [3], were rated as high or unclear risk of bias [2].

The authors detail that, “the use of machine learning has great potential to unearth new insights into the workload and injury relationship.”[1] Prediction models may use both causal and non-causal predictors to estimate the risk of a future outcome [4, 5]. Consequently, it is inappropriate to use the included predictors to infer causal relationships between individual predictors and the outcome [6, 7]. Further, the authors state that Shapley values, local interpretable model-agnostic explanations, and partial dependency plots can be used to assist in interpreting cause-effect relationships with machine learning models [8]. These tools assess for associations between predictors and outcomes and, regardless of how these methods are labelled, the popular adage “correlation is not causation” still holds [8]. Importantly, these methods remain explorative, provide post hoc explanations (rationalization), and require confirmatory studies.

Such inaccurate and incorrect interpretations of clinical prediction models are of particular concern. This is because they can lead practitioners to attempt to change injury risk by intervening or manipulating predictor variables under the false assumption of a causal relationship; while these strategies are likely ineffective, they also have potentially harmful consequences for the athlete [4, 5, 9].

While the authors promote balancing dataset outcomes through over and under-sampling [1], this is highly discouraged as ‘balancing’ datasets alters the outcome prevalence, biasing towards overestimating risk.[10, 11] Balancing data without appropriate recalibration can inappropriately impact risk prediction and ultimately decision-making [11]. The authors also encourage creating classification models. Classification models are not recommended as this supersedes clinical and performance decision-making from the model users [11]. Classification models do not allow situational context and assume all situations and individuals have the same risk threshold. Prediction models should be developed and reported as a probability or at least risk score, to allow user interpretation and decisions [11].

The authors report that area under the curve (a form of discrimination), accuracy, sensitivity, and specificity should be used to evaluate machine learning models. These metrics are only a few of the recommended performance values that should be transparently reported [12] and some (such as accuracy) have well-known problems (also in machine learning) [13, 14]. The authors do not mention calibration as a model performance metric, which is the agreement between predicted and actual probabilities [15]. Calibration is always recommended and allows users to evaluate model performance across a range of risks [4, 5, 12]. Calibration divergence at specific risk ranges can alter clinical and performance decisions, even in models with high area under the curve performance [15].

The authors mention limitations of the included research, often only evaluating a single season of injuries for a given team, and how future research should seek to evaluate the models on subsequent seasons of data. Such a statement calls into question the notion of internal and external validation of these included papers. While the models in these papers have only been internally validated, their results provide a high level of optimism but no real understanding of how they may function under different circumstances or timepoints [16, 17]. Without a process of external validation for such models it is impossible to know whether or not they will be useful to practitioners with different clubs, observing different training constraints, under different coaches, and with different athletes who may have different base levels of risk [6, 18].

The purpose of this letter is to provide clarity about an important and burgeoning sports medicine and performance field. Other points that are not examined due to word count include sample size calculations, handling of missing data, data leakage, internal validation over train test splits, and clinical utility through clinical decision analysis and impact studies. Using the highest quality methods with transparency is imperative to protect and improve athlete health and performance, and to reduce the proliferation of conceptual and methodological inaccuracies about prediction model development and interpretation.

## Data Availability

Data sharing is not applicable to this article as no datasets were generated or analysed during the current study.
